# Relationship Between Diaphragm Thickness, Thickening Fraction, Dome Excursion, and Respiratory Pressures in Healthy Subjects: An Ultrasound Study

**DOI:** 10.1007/s00408-024-00686-2

**Published:** 2024-03-23

**Authors:** Toru Yamada, Taro Minami, Shumpei Yoshino, Ken Emoto, Suguru Mabuchi, Ryoichi Hanazawa, Akihiro Hirakawa, Masayoshi Hashimoto

**Affiliations:** 1 Department of General Medicine, Graduate School of Medical and Dental Sciences, Tokyo Medical and Dental University, Bunkyo-ku, Tokyo, 113-8510 Japan; 2 Medicine, Division of Pulmonary, Critical Care, and Sleep Medicine, The Warren Alpert Medical School of Brown University, Providence, RI 02903 USA; 3 Medicine, Division of Pulmonary, Critical Care, and Sleep Medicine, Care New England Health System, Providence, RI 02906 USA; 4 General Internal Medicine, Iizuka Hospital, Iizuka, Fukuoka 135-0041 Japan; 5 General Internal Medicine, Kaita Hospital, Iizuka, Fukuoka 820-1114 Japan; 6 Department of Clinical Biostatistics, Graduate School of Medical and Dental Sciences, Tokyo Medical and Dental University, Bunkyo-ku, Tokyo, 113-8510 Japan

**Keywords:** Diaphragm, Maximal expiratory pressure, Maximal inspiratory pressure, Pulmonary function, Ultrasonography

## Abstract

**Purpose:**

Diaphragm ultrasonography is used to identify causes of diaphragm dysfunction. However, its correlation with pulmonary function tests, including maximal inspiratory (MIP) and expiratory pressures (MEP), remains unclear. This study investigated this relationship by measuring diaphragm thickness, thickening fraction (TF), and excursion (DE) using ultrasonography, and their relationship to MIP and MEP. It also examined the influence of age, sex, height, and BMI on these measures.

**Methods:**

We recruited healthy Japanese volunteers and conducted pulmonary function tests and diaphragm ultrasonography in a seated position. Diaphragm ultrasonography was performed during quiet breathing (QB) and deep breathing (DB) to measure the diaphragm thickness, TF, and DE. A multivariate analysis was conducted, adjusting for age, sex, height, and BMI.

**Results:**

Between March 2022 and January 2023, 109 individuals (56 males) were included from three facilities. The mean (standard deviation) MIP and MEP [cmH2O] were 72.2 (24.6) and 96.9 (35.8), respectively. Thickness [mm] at the end of expiration was 1.7 (0.4), TF [%] was 50.0 (25.9) during QB and 110.7 (44.3) during DB, and DE [cm] was 1.7 (0.6) during QB and 4.4 (1.4) during DB. Multivariate analysis revealed that only DE (DB) had a statistically significant relationship with MIP and MEP (*p* = 0.021, *p* = 0.008). Sex, age, and BMI had a statistically significant influence on relationships between DE (DB) and MIP (*p* = 0.008, 0.048, and < 0.001, respectively).

**Conclusion:**

In healthy adults, DE (DB) has a relationship with MIP and MEP. Sex, age, and BMI, but not height, are influencing factors on this relationship.

**Supplementary Information:**

The online version contains supplementary material available at 10.1007/s00408-024-00686-2.

## Introduction

The diaphragm is the primary muscle involved in respiration. The diaphragm dysfunction is associated with exertional dyspnea, orthopneic breathing, reduced cough strength leading to aspiration risk, and difficulty in weaning from mechanical ventilation [1, 2]. Diaphragm ultrasonography is a non-invasive and convenient method to assess diaphragm function and is useful in diagnosing diaphragmatic paralysis and predicting successful weaning from mechanical ventilation [1, 2]. There are two measurement techniques: one measures the diaphragm excursion (DE) and the other evaluates diaphragm thickness and the change in thickness during respiration (thickening fraction: TF). Common diagnostic criteria for diaphragmatic paralysis include a thickness < 2 mm or a TF < 20% during deep breathing (DB) [1, 3, 4]. Furthermore, DE < 2 cm during quiet breathing (QB) has been proposed as a criterion for diaphragm dysfunction [5].

Diaphragm ultrasonography is becoming increasingly prevalent in clinical practice. Although diaphragm function might be closely related to respiratory function, the specific relationship between respiratory function tests and diaphragm ultrasonography has not been well established [6–8]. For instance, some studies suggested a relationship between thickness at functional residual capacity (FRC) and maximal inspiratory pressure (MIP), whereas others did not [6, 9–11]. Similarly, results are mixed regarding the relationship between DE and maximal expiratory pressure (MEP) or MIP [6, 12–16]. However, these studies had a small sample size. The populations vary between healthy individuals and patients with underlying conditions such as chronic obstructive pulmonary disease or head trauma, making it challenging to integrate the results. A recent systematic review of the relationship between diaphragm ultrasonography and respiratory function tests noted high heterogeneity in terms of study design [7]. Therefore, there is a need for standardized large-scale studies focusing on healthy individuals.

In this study, we conducted pulmonary function tests and diaphragm ultrasonography on 109 healthy Japanese volunteers to investigate the relationship between diaphragm ultrasonography parameters, including thickness, TF, and DE, with the MIP and MEP. Additionally, we explored factors that influenced these relationships.

## Methods

### Study Population and Setting

This study was a secondary analysis of a cross-sectional study of diaphragmatic ultrasonography on healthy Japanese [17]. Healthy adult Japanese volunteers, age 18-year-old or older, were recruited from three facilities in Tokyo, Fukuoka and Kanagawa prefecture. The recruitment period was from March 2022 to January 2023. On the examination day, volunteers provided information about their age, sex, height, weight, smoking history, and medical history. Subsequently, pulmonary function tests were conducted using a Spirometer (AutoSpiro507, Minato Medical Science Co. Ltd.) to measure the percent vital capacity (%VC), forced expiratory volume in one second (FEV1), and forced vital capacity (FVC). Only asymptomatic individuals with %VC ≥ 80% and FEV1/FVC ≥ 70% were included. MIP and MEP were measured twice each, and the better of the two results was used. All diaphragm ultrasonography measurements were performed by physicians certified as instructors in the Point of Care Ultrasound Simulation Course and by trained ultrasonography technicians.

### Ultrasound Measurements of the Diaphragm

The right hemidiaphragm was measured by ultrasonography in a seated position. The DE of the right hemidiaphragm was assessed at the area around the eighth to ninth intercostal space along the anterior to mid-axillary line, where the diaphragm dome is visualized. A phased-array transducer (2.5 MHz) was placed longitudinally and perpendicularly to the chest wall and adjusted to avoid the ribs. The M-mode interrogation line was adjusted to be as perpendicular to the diaphragm as possible, and the difference in the dome’s movement during inspiration and expiration was measured. For the thickness of the right hemidiaphragm measurement, a linear transducer (7.0 MHz) was positioned longitudinally and perpendicularly at the zone of apposition of the diaphragm, near the eighth to ninth intercostal space along the anterior to mid-axillary line. It was then adjusted to avoid the ribs and positioned so that the lung was partially visible at the edge of the screen during inspiration. At this site, the diaphragm thickness during inspiration and expiration was measured. The thickness measurements were conducted in B-mode, with measurement markers placed from the center of the white line on the thoracic side of the diaphragm to the center of the white line on the peritoneal side. The TF was calculated as follows: (thickness at the end of inspiration − thickness at the end of expiration)/thickness at the end of expiration × 100. The TF and DE were measured during QB and DB.

### Statistical Analysis

The patients’ characteristics, diaphragm thickness, TF, and DE are presented as the mean and standard deviation (SD) for continuous data and as counts and proportion for categorical data. Simple and multiple linear regression analyses were used to evaluate the associations between thickness, TF, and DE, and MIP and MEP. In the multiple linear regression analyses, patients’ characteristics including age, sex, height, and BMI were adjusted. We considered *p* < 0.05 to indicate statistical significance. All data analyses were performed using STATA version 17.0 (StataCorp LLC, College Station, TX, USA).

## Results

### Participants

A total of 111 Japanese volunteers were recruited for this study. Of these, two individuals were excluded because their %VC was below 80%, resulting in 109 participants being included. The proportion of male volunteers was 51%, with an average age of 31.8 years, ranging from 19 to 60 years. The average BMI was 22.5 for males and 21.7 for females, which was closely aligned with the Japanese national averages of 23.6 for males and 21.8 for females [18]. Two participants had a history of bronchial asthma but were asymptomatic on the day of measurement and had %VC and FEV1/FVC within the normal range. Patient characteristics are presented in Table 1.

**Table 1:** Participant characteristics

	Total	*n* = 109	Men	*n* = 56	Women	*n* = 53
	Mean	(SD)	Mean	(SD)	Mean	(SD)
Age [years]	31.8	(11.3)	30.3	(8.9)	33.5	(13.3)
Height [cm]	165.3	(9.4)	172.2	(6.1)	158	(6.1)
Body mass index	22.1	(2.8)	22.5	(2.9)	21.7	(2.6)
%VC	96.5	(10.1)	97.8	(9.3)	95.1	(10.7)
FEV1/FVC	85.6	(5.2)	85.2	(4.7)	86	(5.6)
MIP [cmH_2_O]	72.2	(24.6)	84.8	(24.5)	58.9	(16.5)
MEP [cmH_2_O]	96.9	(35.8)	119.1	(33.3)	73.5	(20.1)
Smoking history, n (%)						
Never smoked	94	(86.2)	49	(87.5)	45	(84.9)
Former smoker	12	(11.0)	5	(8.9)	7	(13.2)
Current smoker	3	(2.8)	2	(3.6)	1	(1.9)
Medical history						
Asthma	2	(1.8)	1	(1.8)	1	(1.9)

*SD* standard deviation, *%VC* percent vital capacity, *FEV1/FVC* forced expiratory volume in one second/forced vital capacity, *MIP* maximum inspiratory pressure, *MEP* maximum expiratory pressure

### Diaphragm Thickness, TF, and DE

The mean values of measurements for the diaphragm thickness (FRC), TF (QB), TF (DB), DE (QB), and DE (DB) were 1.7 mm (SD 0.4), 50.0% (SD 25.9), 110.7% (SD 44.3), 1.7 cm (SD 0.6), and 4.4 cm (SD 1.4), respectively. The thickness and TF were measured in all participants during QB and DB. DE could not be measured in one individual during QB and in 10 individuals during DB because the lung overlaid the diaphragm during inspiration. The measurement results are presented in Table 2.

**Table 2:** Diaphragm thickness, thickening fraction, and excursion of the right diaphragm

Parameter	Respiratory pattern	*n*	Mean (SD)		
			Total	Men	Women
Thickness at the end of expiration [mm]	FRC	109	1.7 (0.4)	1.8 (0.3)	1.6 (0.5)
Thickening fraction [%]	Quiet breathing	109	50.0 (25.9)	46.4 (20.8)	53.8 (30.0)
	Deep breathing	109	110.7 (44.3)	113.8 (46.0)	107.4 (42.6)
Diaphragm excursion [cm]	Quiet breathing	108	1.7 (0.6)	1.8 (0.6)	1.6 (0.6)
	Deep breathing	99	4.4 (1.4)	5.1 (1.3)	3.8 (1.1)

*FRC* functional residual capacity, *SD* standard deviation

### Simple and Multiple Regression Analyses

Initially, a linear univariate regression analysis was conducted with MIP or MEP as the outcome variable to examine their relationship with diaphragm thickness, TF, and DE. A statistically significant relationship was observed between the MIP and DE during DB (*p* < 0.001), between the MEP and TF during QB (*p* = 0.048), and the DE during DB (*p* < 0.001). No relationship was found between the MIP or MEP, and thickness or TF during DB or DE during QB (Table 3).

**Table 3:** Results of simple regression analysis for MIP, MEP, and independent variables

Independent variables			Outcome variables			
			MIP		MEP	
Parameter	Respiratory pattern	*n*	Coefficient [95% CI]	*p* value	Coefficient [95% CI]	*p* value
Thickness at the end of expiration [mm]	FRC	109	5.85 [− 5.05, 16.74]	0.290	8.61 [− 7.2, 24.47]	0.284
Thickening fraction of diaphragm [%]	Quiet breathing	109	− 0.12 [− 0.30, 0.06]	0.202	− 0.26 [− 0.52, < − 0.01]	0.048*
	Deep breathing	109	0.03 [− 0.08, 0.14]	0.575	− 0.07 [− 0.22, 0.09]	0.393
Diaphragm excursion [cm]	Quiet breathing	108	0.04 [− 0.75, 0.83]	0.920	0.58 [− 0.57, 1.72]	0.322
	Deep breathing	99	0.85 [0.54, 1.16]	< 0.001*	1.24 [0.79, 1.69]	< 0.001*

*MIP* maximum inspiratory pressure, *MEP* maximum expiratory pressure, *FRC* functional residual capacity, *CI* confidence interval

*p < 0.05

In the multivariate linear regression analysis adjusted for age, sex, height, and BMI, a statistically significant relationship was only observed for DE during DB for the MIP (*p* = 0.008) and MEP (*p* = 0.021). Sex and BMI were statistically significant influencing factors for all parameters in relation to the MIP and MEP. Age had a significant influence on all parameters in relation to the MIP and MEP except for thickness and DE during DB for MEP. Height had no influence on any of the parameters (Table 4 and 5).

**Table 4:** Results of multiple regression analysis for MIP and result of each diaphragm measurement adjusted for independent variables

Independent variables		Outcome variables: MIP					
		Model 1: *n* = 109		Model 2: *n* = 109		Model 3: *n* = 109	
Parameter	Respiratory pattern	Coefficient [95% CI]	*p* value	Coefficient [95% CI]	*p* value	Coefficient [95% CI]	*p* value
Thickness at the end of expiration [mm]	FRC	− 5.26 [− 15.28, 4.76]	0.300				
Thickening fraction of diaphragm [%]	Quiet breathing			− 0.07 [− 0.21, 0.08]	0.352		
	Deep breathing					0.02 [− 0.07, 0.10]	0.669
Age [years]		− 0.40 [− 0.76, − 0.03]	0.033*	− 0.46 [− 0.81, − 0.11]	0.010*	− 0.46 [− 0.81, − 0.11]	0.010*
Female sex (reference: male)		− 21.15 [− 32.78, − 9.52]	< 0.001*	− 19.24 [− 30.84, − 7.64]	0.001*	− 19.99 [− 31.50, − 8.48]	0.001*
Height [cm]		0.12 [− 0.50, 0.73]	0.710	0.15 [− 0.47, 0.76]	0.639	0.12 [− 0.50, 0.74]	0.697
Body mass index		3.48 [1.99, 4.97]	< 0.001*	3.27 [1.85, 4.68]	< 0.001*	3.24 [1.83, 4.66]	< 0.001*

Independent variables		Outcome variables: MIP			
		Model 4: *n* = 108		Model 5: *n* = 99	
Parameter	Respiratory pattern	Coefficient [95% CI]	*p* value	Coefficient [95% CI]	*p* value
Diaphragm excursion [cm]	Quiet breathing	− 0.27 [− 0.91, 0.37]	0.408		
	Deep breathing			0.43 [0.11, 0.75]	0.008*
Age [years]		− 0.47 [− 0.83, − 0.12]	0.010*	− 0.49 [− 0.85, − 0.13]	0.008*
Female sex (reference: male)		− 20.50 [− 31.86, − 9.13]	0.001*	− 12.21 [− 24.31, − 0.11]	
Height [cm]		0.17 [− 0.44, 0.79]	0.584	0.27 [− 0.35, 0.89]	0.385
Body mass index		3.45 [2.02, 4.87]	< 0.001*	3.03 [1.46, 4.60]	< 0.001*

Model 1: Regression analysis model of MIP and thickness adjusted for age, sex, height, and body mass index

Model 2: Regression analysis model of MIP and thickening fraction of quiet breathing adjusted for age, sex, height, and body mass index

Model 3: Regression analysis model of MIP and thickening fraction of deep breathing adjusted for age, sex, height, and body mass index

Model 4: Regression analysis model of MIP and diaphragm excursion of quiet breathing adjusted for age, sex, height, and body mass index

Model 5: Regression analysis model of MIP and diaphragm excursion of deep breathing adjusted for age, sex, height, and body mass index

MIP: maximum inspiratory pressure; FRC: functional residual capacity; CI: confidence interval

*p < 0.05

**Table 5:** Results of multiple regression analysis for MEP and result of each diaphragm measurement adjusted for independent variables

Independent variables		Outcome variables: MEP					
		Model 1: *n* = 109		Model 2: *n* = 109		Model 3: *n* = 109	
Parameter	Respiratory pattern	Coefficient [95% CI]	*p* value	Coefficient [95% CI]	*p* value	Coefficient [95% CI]	*p* value
Thickness at the end of expiration [mm]	FRC	− 9.17 [− 22.74, 4.40]	0.183				
Thickening fraction of diaphragm [%]	Quiet breathing			− 0.16 [− 0.35, 0.04]	0.112		
	Deep breathing					− 0.10 [− 0.21, 0.02]	0.099
Age [years]		− 0.40 [− 0.89, 0.10]	0.114	− 0.50 [− 0.97, − 0.04]	0.035*	− 0.47 [− 0.94, 0.00]	0.048*
Female sex (reference: male)		− 45.93 [− 61.69, − 30.18]	< 0.001*	− 42.12 [− 57.76, − 26.48]	< 0.001*	− 44.58 [− 60.03, − 29.13]	< 0.001*
Height [cm]		− 0.21 [− 1.05, 0.62]	0.609	− 0.14 [− 0.98, 0.68]	0.722	− 0.18 [− 1.01, 0.64]	0.660
Body mass index		4.00 [1.98, 6.01]	< 0.001*	3.64 [1.74, 5.56]	< 0.001*	3.51 [1.61, 5.42]	< 0.001*

Independent variables		Outcome variables: MEP			
		Model 4: *n* = 108		Model 5: *n* = 99	
Parameter	Respiratory pattern	Coefficient [95% CI]	*p* value	Coefficient [95% CI]	*p* value
Diaphragm excursion [cm]	Quiet breathing	0.02 [− 0.87, 0.91]	0.963		
	Deep breathing			0.52 [0.08, 0.97]	0.021*
Age [years]		− 0.52 [− 1.01, − 0.03]	0.038*	− 0.46 [− 0.96, 0.05]	0.074
Female sex (reference: male)		− 44.14 [− 59.86, − 28.43]	< 0.001*	− 33.25 [− 50.13, − 16.37]	< 0.001*
Height [cm]		− 0.20 [− 1.05, 0.65]	0.65	< 0.01 [− 0.86, 0.87]	1.00
Body mass index		3.68 [1.71, 5.65]	< 0.001*	3.04 [0.85, 5.22]	0.007*

Model 1: Regression analysis model of MEP and thickness adjusted for age, sex, height, and body mass index

Model 2: Regression analysis model of MEP and thickening fraction of quiet breathing adjusted for age, sex, height, and body mass index

Model 3: Regression analysis model of MEP and thickening fraction of deep breathing adjusted for age, sex, height, and body mass index

Model 4: Regression analysis model of MEP and diaphragm excursion of quiet breathing adjusted for age, sex, height, and body mass index

Model 5: Regression analysis model of MEP and diaphragm excursion of deep breathing adjusted for age, sex, height, and body mass index

MEP: maximum expiratory pressure; FRC: functional residual capacity; CI: confidence interval

*p < 0.05

## Discussion

This study investigated the relationship between ultrasonographic findings (diaphragm thickness, TF, and DE) and pulmonary function test findings (MIP and MEP) in healthy volunteers. Additionally, it explored which factors influenced these relationships. This represents the largest-scale study to date conducted on a healthy population. [6, 7, 9–11] Results from the multiple regression analysis, adjusted for age, sex, height, and BMI, demonstrated a statistically significant relationship between the DE (DB), and the MIP and MEP. Thickness, TF (QB), and TF (DB) had no relationship with MIP and MEP. Although previous studies reported a relationship between the thickness, TF, and DE with MIP and MEP in healthy individuals, all were univariate analyses, and none had been adjusted for variables such as age, sex, or BMI [6, 11, 19]. However, previous studies reported relationships between the diaphragm thickness, TF, and DE with age, sex, and BMI [4, 6, 20–23]. To accurately evaluate the relationship between various diaphragm ultrasonography parameters and the MIP or MEP, it is essential to adjust for these factors. In this study, the univariate analysis indicated a relationship between the TF (QB) and MEP, although it was not observed in the multivariate analysis. Sex and BMI were statistically significant influencing factors in the relationship between thickness, TF, and DE, and MIP and MEP, with female sex having a negative influence and BMI a positive influence. Therefore, future studies that compare results between groups with significantly different average BMIs, such as Asians and Western populations, must consider the background BMI in their assessments.

### Ultrasonographic Findings and MIP

In the current study, no relationship was found between the thickness (FRC) and MIP. This finding is consistent with previous studies involving 13 healthy individuals [11] and 64 healthy individuals [6]. Although these studies had small sample sizes, our analysis with 109 subjects also did not demonstrate a relationship between the thickness (FRC) and MIP. Two other studies that reported there was a relationship between the thickness (FRC) and MIP had sample sizes of 36 and 24 participants, respectively. Thus, our study sample size of 109 subjects can be considered sufficiently large in comparison [9, 10]. Therefore, it is unlikely that the reason our study did not show a statistically significant relationship is due to an insufficient sample size. The first study included 36 participants, of whom 15 were weight-lifters and 3 were children [9] and the second study focused exclusively on 24 individuals aged 65 years and over [10]. Thus, the participant profiles in these studies were not representative of a general healthy population. In our findings, age had a negative influence and BMI had a positive influence on the variables (*p* = 0.033, *p* < 0.001). Considering these facts, the two previous studies did not adjust for BMI when assessing the thickness of the diaphragm in weightlifters, who are presumed to have a thicker diaphragm than the general population, nor did they adjust for age when considering the elderly, who are presumed to have a thinner diaphragm, potentially affecting the outcomes [9, 10]. Although thickness (FRC) might be an indirect indicator of muscle mass, this might not directly reflect muscle strength. Therefore, diaphragm thickness may not be directly applicable for predicting respiratory muscle strength [6, 24].

TF, either in QB or DB, had no relationship with MIP. Few studies have examined the relationship between TF and MIP in healthy individuals. A study of 10 children with an average age of 11 years by Ho et al. reported a Spearman’s rank relationship coefficient of 0.64 between the TF and MIP [19]. In our multiple regression analysis, age had a negative influence on the relationship between TF and MIP. Because the results of the study by Ho et al. were not adjusted for age, it is possible that age had a significant impact on the study findings. However, it is unclear whether children and adults can be discussed in a similar fashion. Additionally, a study by Cardenas et al. [6], involving 64 healthy individuals, reported a positive relationship between the TF (DB) and MIP. The average BMI was 26.1 for males and 25.5 for females, which is higher than our study (22.5 for males and 21.7 for females). BMI had a positive effect on the relationship between TF and MIP in our result. However, the study by Cardenas did not adjust for BMI when performing the analysis, which might have influenced their results because the higher BMI in their study might have suggested a relationship between TF and MIP.

DE (DB) had a significant relationship with the MIP (*p* = 0.008), whereas DE (QB) did not (*p* = 0.408). These results are consistent with past studies [6, 12, 15]. However, a study by Dos Santos Yamaguti et al. reported the DE (DB) did not correlate with MIP [14]. That study targeted chronic obstructive pulmonary disease patients, who, compared with healthy individuals, had a markedly reduced DE, which might have influenced the results [14]. DE is a critical factor involved in altering thoracic content volume. A larger DE results in a greater change in thoracic content volume, thereby increasing the negative pressure on the lungs. Consequently, this can lead to an increase in MIP. The lack of a relationship between DE (QB) and MIP and the observed relationship with DE (DB) can be understood from this pathophysiological perspective.

### Ultrasonographic Findings and MEP

No relationships were found between the thickness, TF (QB), TF (DB), DE (QB), and MEP. Given that the diaphragm is primarily involved in inspiration, it is plausible that it does not have a relationship with MEP. However, a significant relationship was observed between the DE (DB) and MEP. The reason for this may be the influence of the MIP and MEP being related. Simple regression analysis and multiple regression analysis adjusted for age, sex, height, and BMI showed a statistically significant positive relationship between the MIP and MEP (both *p* < 0.001) (Supplementary Information 1). These findings might have impacted the observed relationship between the DE (DB) and MEP.

In light of these findings, diaphragmatic ultrasonography could be applied to predict successful weaning from mechanical ventilation. In fact, diaphragm ultrasonography has been used increasingly in intensive care units to predict successful weaning from mechanical ventilation [2, 25]. This involves diaphragmatic ultrasonography during a spontaneous breathing trial (SBT) to assess the TF or DE, and to predict successful weaning. The combined sensitivity and specificity for DE are 0.85 and 0.75 whereas these values are 0.80 and 0.80 for TF during QB [2]. MIP and MEP are also important indicators of successful weaning [26–28], but measuring these in mechanically ventilated patients during routine clinical SBT is challenging. For instance, encouraging patients who are able to communicate to take deep breaths during SBT and evaluating the DE during DB may further increase the success rate of weaning. However, the threshold values for this are unknown, necessitating further research.

This study had some limitations. Because the participants in this study had an average BMI, it is unclear whether these results are applicable to patients with severe obesity. In cases of extremely high BMI, MIP may actually decrease because of factors such as reduced thoracic compliance. Additionally, since the subjects of this study are young, healthy individuals, it is unclear whether the findings can be applied to patients with underlying diseases or to the elderly. Further research is needed in the future.

In summary, this study investigated the relationship between ultrasonographic findings (diaphragm thickness, TF, and DE) and pulmonary function test findings (MIP and MEP) in healthy volunteers. The influence of age, sex, height, and BMI on these factors were also investigated. The DE (DB) was related to the MIP and MEP, whereas thickness (FRC), TF (QB), and TF (DB) had no relationship. Female sex and older age negatively influence the relationship. Higher BMI positively influences, whereas height does not have an influence.

### Supplementary Information

Below is the link to the electronic supplementary material.

Supplementary file1 (DOCX 24 KB)

## Abbreviations

BMI: Body mass index
DB: Deep breathing
DE: Diaphragm excursion
FEV1: Forced expiratory volume in one second
FRC: Functional residual capacity
FVC: Forced vital capacity
MEP: Maximal expiratory pressure
MIP: Maximal inspiratory pressure
QB: Quiet breathing
SBT: Spontaneous breathing trial
SD: Standard deviation
TF: Thickening fraction
TLC: Total lung capacity
%VC: Percent vital capacity
