# Supplementary material for: Relationship Between Diaphragm Thickness, Thickening Fraction, Dome Excursion, and Respiratory Pressures in Healthy Subjects: An Ultrasound Study
Source: Lung. 2024 Mar 23;202(2):171–8. doi: 10.1007/s00408-024-00686-2 (PMC11009751; doi:10.1007/s00408-024-00686-2)
Supplement: Supplementary file 1 — Supplementary file1 (DOCX 24 KB) [file 408_2024_686_MOESM1_ESM.docx]

**Relationship Between Diaphragm Thickness, Thickening Fraction, Dome Excursion, and Respiratory Pressures in Healthy Subjects: an Ultrasound Study**

Toru Yamada, Ph.D.^1^, Taro Minami, M.D.^2.3^, Syumpei Yoshino, M.D.^4^, Ken Emoto, M.D.^5^, Suguru Mabuchi, Ph.D.^1^, Ryoichi Hanazawa, M.Sc.^6^, Akihiro Hirakawa, Ph.D.^6^, Masayoshi Hashimoto, Ph.D.^1^

^1^Department of General Medicine, Graduate School of Medical and Dental Sciences, Tokyo Medical and Dental University, Bunkyo-ku, Tokyo 113-8510, Japan

^2^Medicine, Division of Pulmonary, Critical Care, and Sleep Medicine, The Warren Alpert Medical School of Brown University, Providence, RI, 02903, USA

^3^Medicine, Division of Pulmonary, Critical Care, and Sleep Medicine, Care New England Health System, Providence, RI, USA

^4^General Internal Medicine, Iizuka Hospital, Iizuka, Fukuoka 135-0041, Japan

^5^General Internal Medicine, Kaita Hospital, Iizuka, Fukuoka 820-1114, Japan

^6^Department of Clinical Biostatistics, Graduate School of Medical and Dental Sciences, Tokyo Medical and Dental University, Bunkyo-ku, Tokyo 113-8510, Japan

**Supplementary Information**

Supplementary information 1: Results of simple and multiple regression analysis for MEP and MIP

| Simple and multiple regression analysis for MEP and MIP | | |
| --- | --- | --- |
| **Independent variables** | **Outcome variables: MIP n=109** | |
| **Parameter** | **Coefficient [95% CI]** | **p value** |
| MEP [cmH2O] | 0.52 [0.44, 0.61] | <0.001* |
| Multiple regression analysis for MEP and MIP adjusted for age, sex, height, and body mass index | | |
| **Independent variables** | **Outcome variables: MIP n=109** | |
| **Parameter** | **Coefficient [95% CI]** | **p value** |
| MEP [cmH2O] | 0.44 [0.32, 0.55] | <0.001* |
| Age [years] | −0.24 [−0.52, 0.05] | 0.102 |
| Female sex (reference: male) | −0.83 [−11.40, 9.74] | 0.876 |
| Height [cm] | 0.21 [−0.29, 0.71] | 0.401 |
| Body mass index | 1.68 [0.46, 2.89] | 0.007* |

MIP: maximum inspiratory pressure; MEP: maximum expiratory pressure; CI: confidence interval.

*p<0.05.
